# Do Regulatory Changes Seriously Affect the Medical Devices Industry? Evidence From the Czech Republic

**DOI:** 10.3389/fpubh.2021.666453

**Published:** 2021-04-28

**Authors:** Petra Maresova, Lukas Rezny, Lukas Peter, Ladislav Hajek, Frank Lefley

**Affiliations:** ^1^Department of Economics, University of Hradec Kralove, Hradec Kralove, Czechia; ^2^Department of Cybernetics and Biomedical Engineering, Technical University of Ostrava, Ostrava, Czechia

**Keywords:** risk, patient safety, regulation, innovation, SME, medical devices

## Abstract

**Background:** Within the EU, some of the challenges and perceived risks now facing medical device (MD) developers result from changes in the regulatory framework, emphasizing safety. Therefore, medical technology companies must adopt stricter quality assurance measures so that individual devices can be speedily tracked and retrieved in emergency situations.

**Objectives:** We highlight the challenges and risks faced by the European medical devices industry, particularly those faced by SMEs in the Czech Republic. We address two important research questions: Q1. Do advantages from increased regulation outweigh the additional expenses? Q2. As many MD developers are SMEs, will the new regulatory regime result in some of those companies going out of business and therefore impede future innovation?

**Methods:** The paper focuses on a single case study, with the situation and outcomes discussed in the context of the financial results of a further 50 medical device manufacturers marketing in the Czech Republic.

**Results:** Our findings suggest that the new legislation will result in improved safety, facilitate product recalls, but the cost and administrative burden may be high. The evidence also indicates that some SMEs may be forced to diversify to “non-medical” products, with the inevitable loss of innovative MDs being made available to patients and healthcare providers.

## Introduction

The European trade association for the medical technology industry (MedTech) state that “medical technologies can save lives, improve health, and contribute to sustainable healthcare” (1). The medical health literature (2) states, “Medical devices are used for the diagnosis, monitoring, and treatment of virtually every disease or condition, and include familiar objects such as simple bandages to high-end MRI scanners” regulated by Council Directive (3, 4). European manufacturers now face the new Regulation (EU) 2017/745 to be introduced in 2021. This new legislation not only presents issues concerning health and risk but also for society in general.

Attitudes toward regulations are perceived differently. Concern has been expressed in the literature that the current regulatory regime for medical devices (MDs) is inadequate, biased toward commercial interests, that innovation outpaces the development of regulatory controls, resulting in social and patient risk implications (5–7). de Mol (8) argues (p. 735) that “medical devices are considered to be a cornerstone of medical technology and to enable progress in healthcare for millions of patients.” The current regulatory regime may, however, be putting patients at risk (9). With respect to innovation theories, a strict interpretation of the Porter hypothesis implies that challenging factors in the form of heavy regulations can induce innovation because established technologies get to be replaced by newer, more effective and safer alternatives (10). This may be particularly true of the medical device market, which is highly regulated, and new entrants must fulfill many conditions. These conditions involve mainly safety regulations but also environmental protection requirements, and they affect multiple aspects of the development process: technical, clinical, as well as biological. While the demanding conditions may be seen as an obstacle, they may also prove stimulating for innovation (11).

This industry's characteristics are the exceptionally high innovation potential (11), the above-average number of innovations successfully applied to the market, the high added value of products, and the high export potential (12). The manufacturers of medical technologies have high-level research and development capacities. They are also vocal in expressing their expertise and knowledge toward the continuous development of innovative and accelerated development (13). As a result, such development activities in the Czech Republic often result in products with unique properties, which are considered innovative globally. The research, development, and production of MDs have a long tradition in the Czech Republic (14). In addition to the positive impact on the economic development of the Czech Republic, the development and production of MDs also have a direct positive impact on other sectors, especially the health services sector (13).

The main change under the new MDR regulations focuses on safety and risk reduction, which is to be achieved by strict processes that lead to market authorization (15). Medical technology companies must adopt more stringent quality assurance measures so that individual devices can be speedily tracked and retrieved in emergencies. Among the new requirements introduced by the MDR is creating a unique position to be filled by a candidate with proof of experience in medical device regulations. This person is to be entrusted with managing all matters related to regulatory requirements (16). For SMEs in the Czech Republic, it can be challenging to find an employee with such expertise. Moreover, “to obtain MDR authorization for class III, implantable devices and high-risk class IIb, MedTech companies will be required to present a notified body with a large volume of clinical data that supports their products' clinical performance” (17).

However, from an SME perspective, the new European legislation (highlighted in this paper) may result in a significant proportional increase in costs and an increased administrative burned, resulting in the impossibility for some companies to continue developing new products and, therefore, restrain innovation for the development of MDs.

This paper aims to highlight the challenges and risks faced by the European medical devices industry due to recent legislation changes, particularly those faced by SMEs in the Czech Republic. We address two important research questions:

Q1. Do advantages from increased regulation outweigh the additional expenses?
Q2. As a large proportion of MD developers are SMEs, will the new regulatory regime result in some of those companies going out of business and therefore restrict future innovation?

It is essential to highlight and bring to the literature the perceived risks (as a result of the proposed new legislation) facing SME companies that currently develop innovative MDs.

Firstly, sector analysis provides an overview of the medical device market and MD companies' structure in the Czech Republic. Secondly, the case study of an SME company describes and analyses the situation from the point of view of regulation and the impact on the functioning of this company. Regardless of the company's size, these problems or requirements will have to be solved by every company.

## Theoretical Background

The importance of innovation in the medical device market and the actual impact on society, the market and the economy is often linked to regulations at national and global levels. It is essential to state the context of the current legislative conditions in force and to acquaint them with their possible impacts, which, for example, some authors have mentioned in the past, both negative and positive.

### R&D, Innovation, and Regulation in the Field of Medical Device Development

Success in medical device development is determined by the variables of innovativeness, financial analysis and planning, user input in the development process, and company employees' engagement in the new product development (NPD) goals. It follows that successful product development and placement on the market depends on a complex interplay of factors relating equally to business strategies, technological solutions, human resources, and end-user involvement. Additionally, a survey found that new global innovations accounted for only 4.4 per cent of NPD projects in larger companies and 9.3 per cent of NPD projects in SMEs (18).

The medical device sector is a rapidly developing industry (19) subjected to pressures from all sides. New technologies emerge in fast succession. They are widely publicized, which results in patients increasing demands for the latest inventions that are still in the early stages of development and may require years of honing and testing before they can be introduced to the market. Therefore, the ever-increasing challenge of small high-tech firms (HTSFs) is to keep up with the latest breakthroughs and come up with ways to leverage their potential (20).

Hourd and Williams (21) conducted a case study on a sample of 14 UK-based SMEs operating in the medical device sector. The researchers compared the individual companies' business strategies and practices to determine the factors most important for these enterprises' success. It was found that each stage of the development process presents its peculiar challenges and that all the stages are equally essential to the overall success. This involves obtaining funding, securing partners, recruiting staff, and setting up development milestones in the preparation stage. Further on, success depends on conducting clinical trials, obtaining approvals, launching the product and conducting post-market surveillance.

Numerous obstacles can hinder the success of the enterprise. These include external cost-related issues, such as lack of funding (22), high implementation cost (22, 23) and the cost of verification or certification (22). Furthermore, barriers may present themselves in the form of unethical regulatory authorities (24) and the difficulty of obtaining information (22).

### The Importance of Regulation for Innovation

The manufacturers of MDs cooperate with clinical workplaces to research and develop new devices and then in the application phase. This co-operation is essential for a high level of medical and nursing care. Therefore, modern MDs represent a fundamental and irreplaceable area for contemporary medicine, which needs to be further developed and innovated (25). An essential prerequisite for the use of innovative MDs for increasing the level of the healthcare provided is to create, in particular, optimal legislative conditions for their market launching.

It is difficult to draw any definitive conclusions when it comes to the relationship between regulation and innovation. The association is a highly complex one, and the impact of regulation on innovation is not always immediately apparent. This impact may be manifested indirectly and gradually, such as in subtle shifts in market structure in competition, business strategies and investment priorities (11).

The new legislation's key goal, highlighted in this paper, is to provide a more transparent, efficient and “consumer-oriented” approach and increase patient safety. The effects of regulation on product quality have been frequently researched in the past. While many theoretical models illustrate the relationships between product quality, competition on the market, and price regulation, only a few studies describe the relationship between regulatory policies and technological innovation. However, a positive relationship between heavy regulation and innovation was established in the Porter hypothesis (10), which proposed the innovation effect of introducing innovative environment-friendly processes as a result of heavy environmental regulations. Simultaneously, innovative technologies are cost-efficient, compensating for any costs incurred in achieving compliance with new regulations. Furthermore, innovations stimulated through regulations can be further utilized through patenting, which ultimately serves as a competitive advantage over companies that are not subjected to such strict regulations.

In contrast, studies that illustrate a negative impact of regulations on innovation (26) highlight that hospitals' regulatory obligations significantly slowed down the spread of CT scans, which eventually became more prevalent in individual physicians' offices than in hospitals. Peltzman (27), researching the effects of new FDA regulations on the drug industry, reports that the regulations led to a significant drop in the number of new drugs introduced in the market and that the costs exceeded any potential savings. Finally, some studies (28, 29) confirm that the new FDA regulations resulted in companies cutting down investments in innovation, hence decreasing the number of new drugs introduced.

What will be the possible consequences of the new medical device regulations [(MDR) 2017/745]? Will it support or hinder innovation?

### Regulatory

Medical technologies are characterized by a constant flow of innovation, resulting from a high level of research and development within the industry and co-operation with users. New product development can take from 1 to 2 years in terms of their risk class. Health means are divided into classes I, IIa, IIb, III. The placing on the market of a new or innovated product depends on the complexity of meeting the essential requirements and, above all, on time required for the conformity assessment by the notified body (NB).

The following risk classifications and descriptions are taken from (17).

**Class I** [I (low-risk non-sterile), Is (sterile), Im (measure), Ir (reusable)] – Provided sterile “and/or have a measuring function (low/medium risk) or reusable low-risk Class I devices placed on the market in a sterile condition, have a measuring function or are reusable surgical instruments: assessment of the technical documentation relating only to those specific features of the device, such as sterility, measurement, or reprocessing” [see also (30)].

**Class IIa** (medium risk) “Class IIa devices: Assessment of the technical documentation for at least one representative device for each category of devices” [see also (31)].

**Class IIb** (medium/high risk) Class IIb “implantable devices (except sutures, staples, dental fillings, dental braces, tooth crowns, screws, wedges, plates, wires, pins, clips, and connectors) and Class IIb active devices intended to administer and/or remove a medicinal product: Assessment of the technical documentation for” [see also (32)].

**Class III** (high risk). Class III devices: “Assessment of the technical documentation for every device.”

Within the European Union, the medical device industry, from development through manufacturing to distribution, is subject to EU directives and regulations. The difference between the two is that directives must be embedded in each member state's national legislative system, whereas regulations apply directly. Legislation concerning medical devices (in the Czech Republic, where our study is based) is spread across a number of different directives, decrees and laws issued by the Ministry of Health of the Czech Republic. These documents apply to medical devices in general or to specific types of devices, such as active implants or *in vitro* diagnostic devices. As an example of legislative conditions, the European manufacturers face the new Regulation (EU) 2017/745 (33) of the European Parliament and the Council on Medical Devices (MDR). Currently, MDs in the Czech Republic are regulated by Act No. 268/2014 Coll. Like the national laws of the other member states of the European Union, this law incorporates Council Directive 93/42/EEC (MDD) on MDs (34).

After placing on the market, MDs must be monitored, and their clinical benefit and, where appropriate, the risks arising from their use must be continuously evaluated. According to Article 10 of the MDR, the manufacturer must set up a so-called proactive way to establish, document, implement, and periodically update the risk management system or conduct a clinical trial under the requirements of Article 61 of the MDR, including post-market clinical follow-up (PMCF) (35). The PMCF is a requirement included in the current MDD, allowing manufacturers to start PMCF studies before the end of the MDD. The clinical data from these studies will be used for MDR clinical trials, thus partially avoiding costly clinical trials. However, manufacturers often have only a post-market surveillance (PMS) process, which they understand as a summary of sales and production; the collection of clinical data is only marginally included here. For this reason, manufacturers lack clinical data that they could rely on in their MDR clinical trials, which can mean high financial demands, not only for the implementation of clinical trials.

The factors mentioned above highlight the European MDs industry's challenges due to recent legislation changes, especially by SMEs. MDR was due to come into force in May 2020, but due to the COVID-19 situation, it has been postponed until May 2021. This gives manufacturers more time and the opportunity to start taking steps to facilitate the transition to MDR. Even though the certificate will be issued and supervised according to the MDD until the end of the transitional period, manufacturers will already be obliged to meet the requirements of the MDR.

### General View of the Medical Device Industry, Specifically in Europe

The development of a new medical device typically starts in a small, innovation-driven company. Small companies tend to stand at the beginning of technological progress in medical devices for several reasons (36). Small-sized companies move forward faster because they are easier to manage than large corporates. The inventor and innovator is often the leader and decision-maker, unlike in larger companies, where research, leadership, and management form different levels. With the innovator and executive in one person, it is significantly easier to make informed decisions and assess possible risks.

This fact is also supported by the number of companies and the structure of the market in Europe. There are 25,000 MedTech enterprises in Europe, 95 per cent of them being SMEs (37, 38). These enterprises are at the greatest risk of exiting the market because administrative costs are often too high for them.

The Czech market is still relatively small compared to other European markets. Demand for medical devices in the Czech Republic in 2016-2020 is relatively constant. In 2016, market growth was 1.3%. In 2019 it was estimated at 2%. Demand for medical devices in the Czech Republic by type of device is highest in areas: Catheters, cannulae and needles, orthopedic and fracture appliances, Medical furniture, Electro-diagnostic equipment, and medical instruments and appliances (39).

The Czech market's opportunities lie in the aging population connected with chronic diseases; there is also demand for innovative products that improve efficiency and health outcomes, such as mini-invasive surgery systems, digital image processing, or home-care equipment (40).

## Research Methodology

This paper is partly based on a single case study, considering that a “Case study is the study of the particularity and complexity of a single case, coming to understand its activity within important circumstances” (41). The popularity of case study research is increasing, especially in corporate research, as case studies can provide insights that might not be achieved with other approaches. However, “The important issue is to have a clear objective, involve the right people and have access to the right information” (42). Case studies can focus on a single case or several cases. There are no formal requirements in a multi-case study as to a minimum number of cases required, and neither is there a requirement of a random selection of cases (43). Therefore, a case study may not be representative since the researcher simply examines such material as is available. Although generalizations from a single case study may be limited, this does not distract from their importance. “Case studies can be used to explain, describe or explore events or phenomena in the everyday contexts in which they occur” (44). Analyzing a particular case in detail may prove to be helpful in discovering and describing cause-and-effect relationships and eventually estimating possible tendencies that may apply to similar cases. This research follows the main steps in preparing and conducting a case study, including successively identifying a specific case, collecting data, interpreting data, and drawing conclusions (45). On top of that, we have performed the industry analysis in the Czech Republic to assess how representative is the case of the selected company in question, medical device producers, which further enhance the usability of the performed case study.

### Design

Our research is based on a single case study supported by data from a further 50 companies. The case study SME company is a representative of Czech manufacturers, listed in the Register of Medical Devices, which since 2015 is a unified system for comprehensive data management in medical devices in the Czech Republic (46). We chose an SME company focusing on the production of several types of MD class IIb, including pacemakers. The fact that many studies confirm the importance of SME companies played a role in selecting the company for our detailed case study. Initially, we looked at the register of the Association of Manufacturers of Medical Technology (47), the total number of 140 companies available. We excluded *in vitro* Diagnostic (IVD) manufacturers (these companies are not affected by the examined change in legislation) and distributors. Sixty-eight companies remained for further analysis. In the next step, economic data were found in the database of Albertina companies' Economic data (48) (profit, revenues and number of employees) were not available for all companies. Our final set contains 53 companies in total (Table 1).

**Table 1:** Number of MD manufacturers by size of company.

**Company category**	**Number of companies**	**The average number of active MDs in the registry**
Micro	13	19.25
Small	18	44.75
Medium-sized	16	63,62
Large	6	57

Generally, our study was performed in the following steps:

- Sector analysis based on data of medical device companies in the Czech Republic to;
  ◦ A list of MD companies was used from the register of the Association of Manufacturers of Medical Technology.
  ◦ For each company information of the production of medical devices according to the risk classes available in the database the National Register of Medical Devices (available at: https://eregpublicsecure.ksrzis.cz/Registr/RZPRO/) Class I-III were included;
  ◦ For the included companies, economic data were added using the Albertina database (48), and economic analysis was done;
- Case study elaboration to show possible scenarios of the impact of new legislation.

### Data

We obtained a sizable dataset from accounting statements and internal company information obtained directly from the company's representative. Our dataset covers the period of 2002–2018, containing a total of 36 variables. Information about company revenues corresponding to the various product classes or services is present, alongside cost categories: salaries, material costs, and specific costs directly related to MD developments and their continual market approval (certification costs). Total revenues, costs, and gross profit are also included.

The dataset is, however, not without its limitations. Since it covers a relatively long time period, many accounting standards changed over the analyzed period; namely, the method for reporting employee numbers (given by full-time vs. part-time contrasts in reporting) changed three times. This made it difficult to obtain comparable data for the entire period.

The company representative was repeatedly interviewed, and the selected financial variables (Table 2) were refined and interpreted. Additionally, an interview was conducted in May 2019 to determine the company's strategy in the reporting period.

**Table 2:** Monitored variables.

**Revenue from the resale of consumable material**	**Purchase of material**	**Cashflow**
Sales of safety technical control (STC)	Purchase of material for resale	Foreign sources (“debt”)
Number of performed STC	Purchase of material for product	Goods (stock of products for sale and material for resale)
Revenue for product	Average adjusted employee count	Stock of material
Number of sold pieces (product)	Employee wages total	Total value of stored stocks (products and material)
Sales of own products and services	Purchase of services, total	Net Capital
Revenue for total sales	Purchase of services, product certification	Subsidy granted by the Ministry of Industry and Trade for the development of a cardio stimulator
Total revenues	Purchase of services, system certification	Subsidy granted by the Ministry of Industry and trade for marketing
Profit before taxes	Total costs	Costs of marketing activities and international exhibitions (fairs)

We used IBM SPSS version 24 was to forecast the main variables for the future company development scenario. SPSS Time Series Modeler was used to select the most appropriate method (exponential smoothing, univariate autoregressive integrated moving average and multivariate) based on the best fit to the historical data (49).

## Results

Innovation in the field of MDs is undertaken by SME companies. Therefore, sector analysis which shows the medical device market in the Czech Republic, is done. Then possible scenarios of the impact of new regulations are shown in a single case study.

### Analysis – Part 1 - Medical Device Industry in the Czech Republic

The major problem of MDR implementation is in the field of innovation. Specifically, most innovative research in MDs is not undertaken by big companies but by SMEs (37, 38). “Such companies usually focus on the production of a small number of products, meaning their margin does not allow them to pay for all the necessary costs connected with MDR compliance” (11). The estimated costs for SMEs to launch a new class III MD will be between EUR 1–4 million or EUR 7–28 million if the device must fulfill the centralized pre-market authorization procedure requirements. A specific expert estimate associated with the legislative conditions is given in Table 3.

**Table 3:** Approximate costs related to the development of MDs (EUR).

		**Inactive**	**Active**		**Active/implants**		**Comments**
		**I**	**I**	**IIa**	**IIb**	**III**	
Time of development [year]		1	1	2	2	2	
Employees	HW	0	17,771	35,542	35,542	35,542	With a salary of 1,500EUR per month
	SW	0	17,771	35,542	35,542	35,542	With a salary of 1,500EUR per month
	other	35542	17,771	71,085	71,085	71,085	With a salary of 1,500EUR per month
Sterilization validation		0	0	1,851	1,851	1,851	
Measurement validation		0	1,851	1,851	1,851	1,851	
Tests el. Security		0	3,702	5,553	5,553	5,553	Not for implantable ones (joints, for stimulators)
EMC		0	1,851	3,702	3,702	3,702	EMC is not implantable (joints, it is a stimulator), but again they have a higher price for biological evaluation
Biological evaluation		1,851	1,851	5,553	5,553	5,553	Based on type of MD
Clinical evaluation		740	740	1,851	3,702	3,702	If the clinical trial is based on a clinical trial, even millions
Type test		740	740	3702	5,553	5,553	
CE Conformity Declaration		0	0	7,405	9,256	11,107	Abroad: notified body: it is IIa - 20 thousand €; IIb - 25 thousand €, III - 30 thousand €, plus supervision every year about 5 thousand €. According to the MDR, it is expected that it should be up to 25% more.
QMS		5,553	5,553	5,553	5,553	5,553	
PMCF		After placing a product on the market, the cost depends on the type of product, its use and the complexity of the study itself					
		(large price range); estimate for IIb is part of the Appendix.					
**Total**		44,428	69,604	17,9193	184,746	186,598	

The above data are an overall estimate for the MD market. However, there are no data available on the impact of new legislation on business results (50). Therefore, we have prepared our specification based on several sources. We have selected companies listed as members of the two MD producer associations in the Czech Republic for our analysis. Our final list consists of 50 companies whose data were relevant and available. See section Design for detailed information. Data are displayed in Table 4.

**Table 4:** Medical device market in the Czech Republic – MD type, production, economic indicators.

**Number of employees**	**Number of firms**	**Average number of active MD in the national MD list**	I*	IIa*	IIb*	III*	**Average weighted profit share in revenues [% of revenues]**	**Expected increase of certification costs as percentage of revenues**
1,500–1,999	1	9				9	10.50%	0.03
1,000–1,499	1	34	34				11.31%	0
250–499	3	14	5	3.5	0.8	1.3	14.04%	0.1
200–249	2	9	1,5	0.5	7	0	15.52%	0.25
100–199	8	34	12	0.8	8.1	5.8	5.42%	1.16
50–99	6	20.2	8,8	5.7	2.7	3	9.96%	0.86
25–49	9	21	17	0.9	1.9	0.8	6.04%	0.78
20–24	4	9.25	3.3	0.5	3.8	1.5	4.69%	2.02
10–19	5	14.5	3.2	1.2	5.6	0	2.12%	5.63
6–9	7	10	6	2.1	1.4	0.9	−16%	5.3
1–5	4	9.25	0	1.3	5	3.3	−169%	10.13

* *Average sum(count) of MD's in a given safety class and group of companies*.

Increases in certification costs for conformity assessment were quantified by a domain expert to be approximately 2.5 points multiple of the current costs, except the MD of safety class I, where there are no increases expected.

We used a combination of data regarding revenues, profit, and the number of MDs in each company's safety classes. Estimates of the increase in certification costs were obtained by multiplying the number of MDs in each respective safety class by the expected rise in certification costs for that class. For companies having multiple MD in the same safety class, coefficients were used to lower estimated certification costs reflecting the fact that producers often certify MD in larger batches (coefficients used were 0.17 for safety class I; 0.26 for class IIa; 0.27 for class IIb and 0.3 for class III). Estimated certification cost increases were further compared with total revenues to obtain a percentage share in revenues to create a reasonable estimate of the significance of this increase for the company. Column 5 in Table 4 displays these results for the groups of companies in our set.

**Figure 1:**
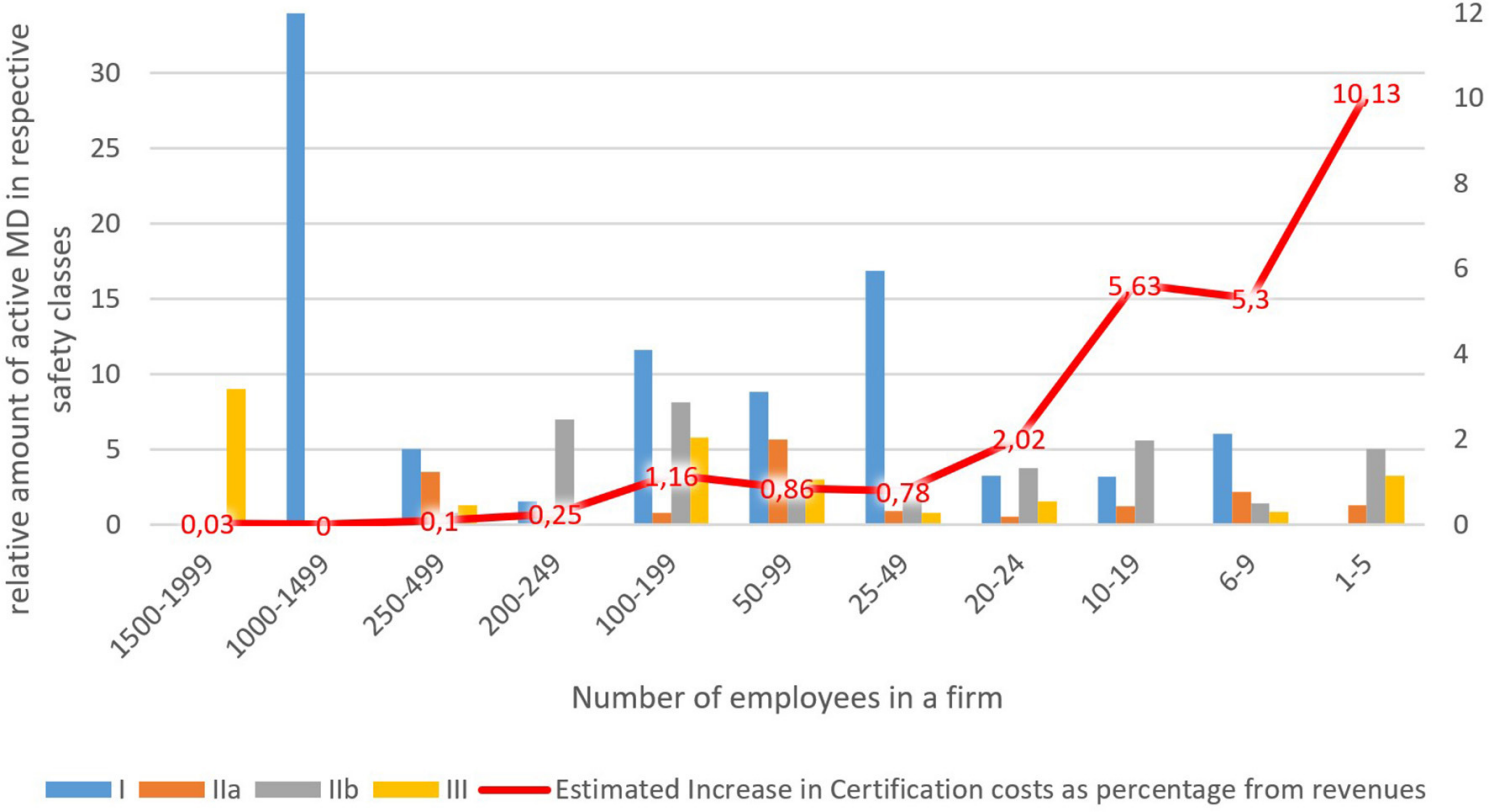
Certification costs.

Results show clearly that the estimated burden of certification cost increases falls disproportionately on small and micro-companies. This is because they have a relatively high share of MDs in higher safety classes, IIb in particular, for which certification costs are quite considerable, but total revenues are relatively small. On top of that, Table 4 shows that profit margins are increasing in line with the company size. The smallest companies, on average, are even recording losses. The red curve in Figure 1 depicts the decreasing estimated increase in certification costs as the percentage of revenues. It is higher than the profit margins for the lower three groups of companies (1–19 employees, 16 companies total) with a relatively small estimated impact on the larger companies (20–199 employees, 27 in total) and negligible impact on the even larger companies (200–999 employees, 7) where it declines significantly to below one per cent of revenues. Only the group of companies with the number of employees in the range of 100–199 show a slight divergence from the trend. This can be explained by the fact that companies in this group have the highest average amount of registered MD's (34) concentrated in the safety classes IIb and III.

### Analysis – Part 2 - Case Study

Our case study is based on an SME (Mediatrade – pulse generators) operating and regulated in the Czech Republic. In the context of the change in legislative conditions in the European Union, the company's crucial concern is related to its economic development under the new legislative conditions.

#### Company Characteristics and Strategy

The company's primary focus, which was established in 1994, is external pacemakers' EPG 10 production and medical devices of risk class IIb. However, it is relatively new to the MD industry, having entered the field in 2012. Secondary activities include the sale of materials for usage in the areas of gastroenterology, specially boot sets for endoscopic stents (guiding, integral and pusher), disposable injector varices; double and triple lumen catheter for ERCP, endoscope catheters, guidewires, Nazo-biliar catheters. In area of cardiology it produce device, cardiostimulation catheter, loader sets, accessories, neurostimulation.

Innovation plays a large part in its product development strategy. Since the company's inception, it launched the first-generation analog products, which it innovated into digital form in 2013, launching its second-generation pacemaker. Based on market demand, the third-generation product with biphasic stimulation impulse was launched in 2017.

Gradually, with increased production, the domestic market was largely saturated (in particular, regional hospitals were fully secured by Mediatrade). The company also provides service for each device in the form of an annual safety check (the historical revenue trend is shown in Supplementary File 1B. Expansion to foreign markets has partially been achieved.

In their business activities, various development problems relating to certification and legislative have been addressed. The company also faced complications in product testing or testing in laboratories for compliance. Another challenge was the compilation of documentation for the new product to be satisfactory in the Czech Republic's legislation. The solution to these problems was partially achieved, in the early years, through certification using external assistance. In later years, professional activities were also dealt with by recruiting external experts.

The product's price was determined based on the market competition with the maximum target price of CZK 40,000. This was because hospitals, if interested in the product, did not have to issue a tender for the product up to and including CZK 40,000. This resulted in reduced administrative costs for hospitals.

#### Economic Indicators of the Company

Mediatrade's financial results show long-term growth between 2002 and 2018, with partial declines in 2007, 2012, and 2017. The revenues follow the development GDP from 2011 to 2017. Despite the economic crisis between 2007 and 2009, the company registered rising revenues (Supplementary File 1C. This corresponds to the healthcare and pharmaceutical industries' performance in the Czech Republic, which did not show a decline in the added value over this same period.

The company has a high debt ratio and a long turnover of inventory and receivables. Its current liquidity points to the ability to repay its short-term liabilities (Table 5). Mediatrade has a value that is more typical for immediate liquidity, that is, very low. The profitability of less than half a percentage point and other indicators adds to an unfavorable economic prospect under the current economic and legislative conditions.

**Table 5:** Economic indicators in 2017.

**Debt 77.41 percent**	**The share of receivables in current assets 144.00 percent**	**Return on EBIT 1.31 percent**
Inventory turnover time 55.00 days	Current liquidity 0.37	Increase/decrease in sales in−13.74 percent
Receivables turnover time 859.00 days	Return on equity 0.53 percent	Average monthly salary 1,519 EUR/month

*Converted at the historical rates for the given years; data are from https://www.kurzy.cz/kurzy-men/historie/EUR-euro/2008/*.

#### Expected Development

To assess the company's possible future development, we have defined four scenarios based on the forecasts of three key variables – revenues for consumption material, amount of performed pacemaker safety – technical controls (STC), and the number of products sold. The analysis of consumption material revenues with exponential smoothing and the prediction of growing revenues resulted in EUR 114,708, EUR 120,760, and EUR 126,813 for 2020, 2021, 2022, respectively. This is based on the historical data for the same variable with observed growth of roughly 150% in the last 10 years and represents a simple trend extrapolation, i.e., analyst estimate. The level of uncertainty concerning this estimate is quite considerable, at around 50% of the forecasted mean value (Upper confidence limit of 148 118 EUR, lower confidence limit of 81 298 EUR on the 95% confidence level for the year 2020). Based on historical data for the amount of performed STC's (usually performed once a year for each device to verify that it functions correctly, i.e., output signal precision is within defined bounds), the SPSS Time-series modeler selected ARIMA (0,0,0) was used for this data series, which equals an interpolation data series in terms of its mean value. In terms of model fit, R-squared was negligible. The prediction does not seem helpful, and consequently, it was not used for further analysis. Fortunately, we know that the amount of performed safety – technical controls each year – is determined by the total number of pacemakers in the Czech Republic hospitals. Mediatrade already reached a cap of 270 active pacemakers in Czech hospitals, so the constant forecast of the 342 performed STC is relevant for the Czech market, given current safety regulations regarding this MD's maintenance. For the last variable, the number of sold pacemakers, the simple exponential smoothing method was used, with a constant predicted value of 48 pieces sold each year. This again corresponds relatively well to our expectations because Mediatrade has already captured the Czech market, and any further growth in product sales may have to come from foreign markets. It is necessary to point out that the trajectories mentioned above of the three primary sources of revenue for the company do not represent exact statistical projections but more so analyst estimates, based on the knowledge of the company inner workings and processes accompanied by an understanding of the Czech market for this particular kind of MD. Costs were calculated using historical data for the corresponding categories; for details, please see the collected data on Mediatrade in Supplementary File 2.

**Figure 2:**
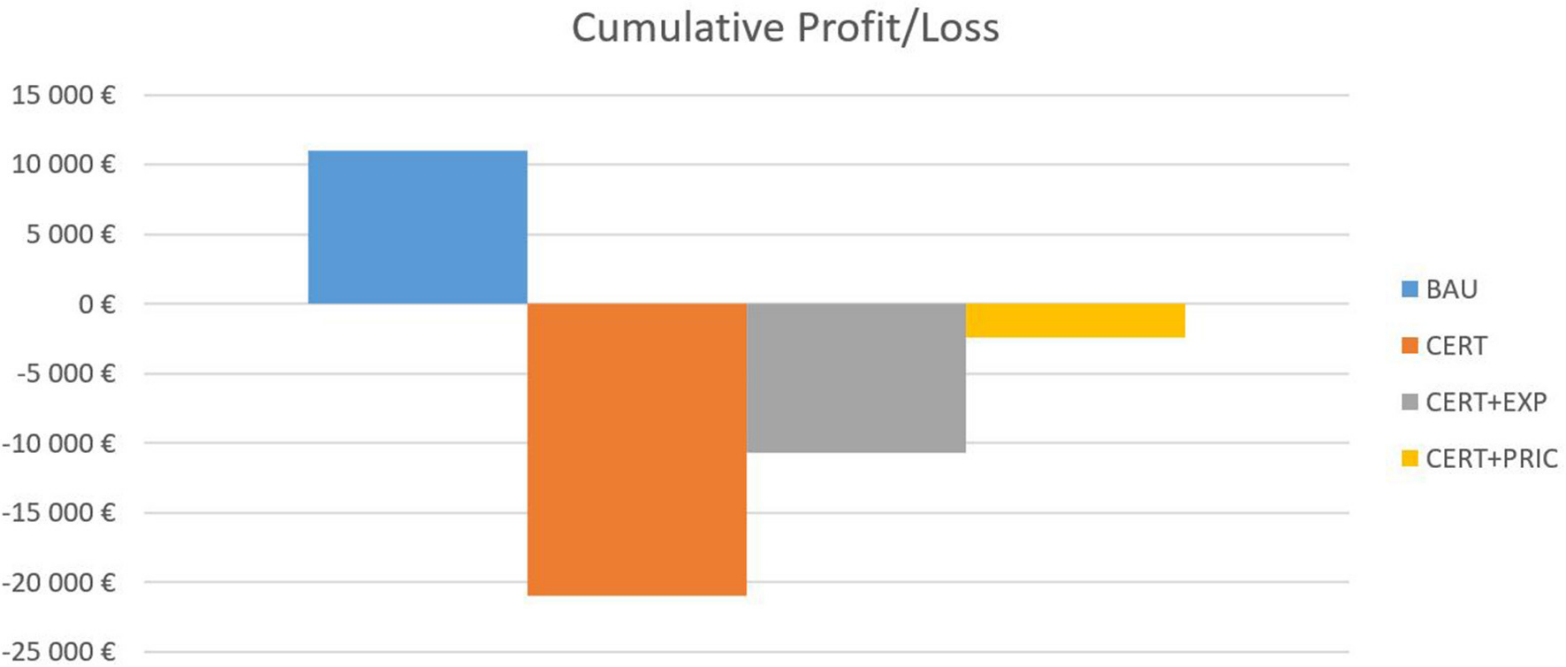
Cumulative profit/loss.

The first scenario is the business-as-usual scenario (BAU), in which there are no significant changes regarding the costs related to MD certification and the sales volume is based on the forecasts explained in the previous paragraph. In the second scenario, the company is faced with increased certification expenditures (CERT) to maintain its MD sales on the Czech medical market. Two following scenarios are based on possible mitigation strategies the company could adopt to offset impacts of CERT scenario – CERT+EXP based on a pro-export company orientation, where it expands on foreign markets with the help of increased marketing expenditures, and CERT+PRIC, where it tries to mitigate the increased certification costs through an increase of 15 per cent in the price of its services and products. We assume inelastic demand for the MD, so the predicted volumes of the units sold and service check-ups decrease by 7.5 per cent overall. The results of this exercise are shown in Figure 2.

Under the BAU scenario, the company generates a small profit over the 3-year forecast period. However, once the increased certification expenditures are included in the CERT scenario, the firm incurs a significant loss, which further impairs its financial standing (EUR 195,160 in debt in 2016). The first mitigation strategy, CERT+EXP, somewhat improves the situation. However, the company still incurs a loss, as the increased volume of sales abroad requires higher marketing expenses and slightly higher employee costs. The last strategy, CERT+PRIC, is the closest to zero, with a small loss of EUR 2,444.

**Figure 3:**
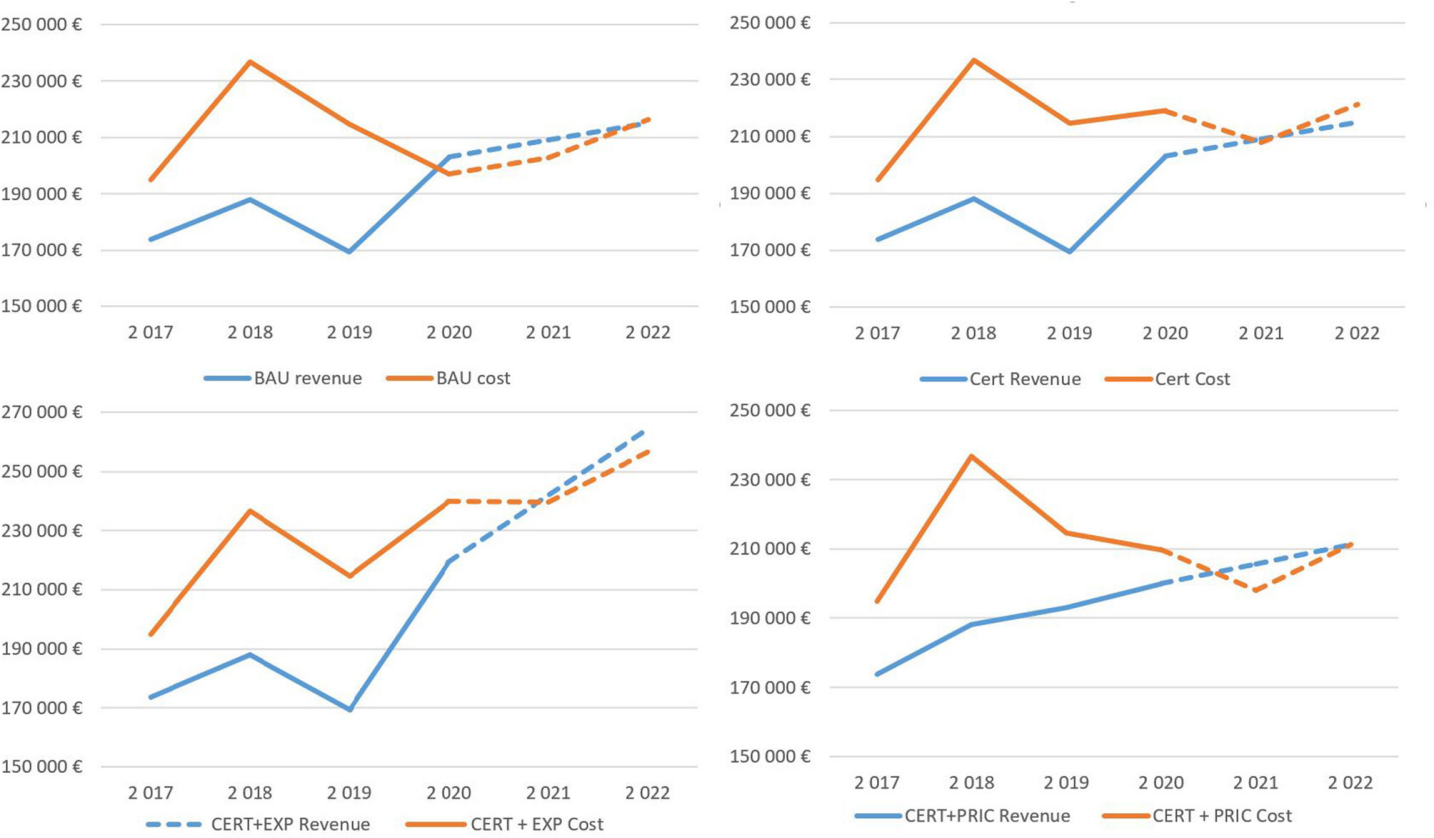
Development of revenues and costs under the simulated scenarios.

Regarding identified uncertainty around forecasted sales of consumption material, the risk is disproportionally skewed to the downside of the forecasted mean value in the post COVID-19 world. A lower confidence limit for the first forecasted year represents a decline of EUR 27,649, enough to wipe out the whole profit in the BAU scenario and significantly deepen the losses in the rest of the scenarios. Also important is the distribution of the results over time due to cashflow consideration; the temporal distribution is depicted in Figure 3.

The first 3 years are based on the historical data obtained from financial statements, and the simulation results are presented for 2020–2022. In all scenarios, losses are concentrated in the first simulation year, but in the two final scenarios, the firm generates a slight profit, except for the BAU scenario.

## Discussion

Previous studies [see, for example, (7)] highlight the need for regulatory change. The current regime appears to be biased toward MD producers and not the end users' safety and risk assessment. Kent and Faulkner (5) argue (p. 189), “that there are weaknesses in the regulation of medical devices and that commercial interests have dominated regulatory policy,” and (p. 191), “The processes of innovation outpace the development of regulatory controls.” There has also been concern expressed about the increasing number of MD recalls (2). Leiter and White (6) argue that “medical devices are increasingly being implanted in human bodies, constituting manufactured risks.”

One of the biggest problems for companies appears to be in acquiring clinical data, which will enable them to succeed in the conformity assessment or certification process, according to MDR 2017/745. Until now, MDD 93/42/EC requirements were perceived differently; the MEDDEV recommendations specified them. However, they were still recommendations. Within the MDR 2017/745, it is no longer a recommendation but a regulation. Thus, there is an obligation for manufacturers to demonstrate clinical data. However, it is not entirely clear whether clinical data from clinical trials or data from PMCF studies will suffice. Manufacturers believe that a well-designed PMCF process will provide them with sufficient quality clinical data to demonstrate their product's clinical safety. However, the rightly set PMCF process is an intervention in the economic situation of the company. It is the right time for manufacturers to decide what changes they will make to their processes and business model to avoid the financial impact on their business.

As the new regulations come into effect, 2021 will be a challenging year for many companies in the MD market. The legislative changes aim to make MDs safer for patients throughout their lifetime. For companies, this means additional control costs, even after an MD has been launched and also new clinical trials for MDs with a lower risk class than in the past.

From our research, it is clear that SMEs will be most affected by the change in legislation. Larger companies are better positioned to adapt to legislation changes because they already have a regulatory department to monitor and update the legislative requirements for their currently manufactured medical devices. Article 15 of MDR 2017/745 specifies that a manufacturer is required to have at least one person within his organization demonstrating his expertise in regulatory affairs, but at the same time admits that small firms may have such a person employed externally. From this point of view, the response to legislative changes in small companies will not be as dynamic as in companies that have established an internal regulatory department. These companies will be forced to make many changes that will impact their economic indicators.

Our market analysis shows that the larger the company the lower the percentage change in increased costs caused by the MDR new requirements. In our dataset, it was also apparent that, in general, micro-cap companies are specialized in the production of a few MDs in higher safety classes. Therefore, they will be disproportionately affected by new regulations, estimates of increases in certification costs as % of revenues are height. Larger companies focus on the large-scale production of class I MD's where it is harder to compete by small companies due to the economies of scale. That leads to specialization on MD's in more demanding safety classes, which will, unfortunately, be harder hit by increases to certification costs. The above-mentioned leads to a potentially threatening situation for SME's. The performed sectoral analysis further showed that the results obtained in the case study of Mediatrade are relevant in the Czech Republic case, as micro-cap companies (see Figure 1) share many of the same characteristics in general.

Overall, there is another problem in the medical device market, corruption. According to Transparency International's Index, the Czech Republic is one of the countries with a higher level of corruption. In the CR are identified illegal and non-standard methods of tendering and vendor lock-in Competitive Procedures Without Negotiation (CPWN). It allows to get the contract to a single bidder without a competition (Act 134/2016 Coll., § 63). In its reports, the Supreme Court repeatedly draws attention to CPWN's practices in securing public procurement, including at the level of ministers and institutions responsible for the purchase of medical devices and equipment. In the years 2011-2016, the share of these contracts ranged from 31 to 62% of their total volume. A common problem with non-standard methods in the health sector is the vendor lock-in, it means propriety lock-in or customer lock-in that makes a customer dependent on a vendor for products and services. Then the contracting authority often tends to compete for long-term contracts in closed procedures, for higher prices on the account of public funds. One of the specific examples in the field of healthcare is the creation of a cartel for the supply of medical equipment and modern technologies to the Czech hospitals (50). There is also pressure from the pharmaceutical and medical device industry toward the use of more profitable products. There is no definition of the standard health care covered by the insurance as guaranteed by the law. It happened in the past that the regulation was *ad-hoc* and usually even retrospective (51). This fat is confirmed by the European Research Centre for Anti-Corruption and State-Building (ERCAS), the Czech Republic has been one of the most successful Centre and Eastern European countries in the fight against corruption in all areas, including the healthcare sector. Nowadays, its administrative simplicity and transparency are comparable to European standards as a result of the harmonization of laws within the European Union (52).

## Conclusions

Concerning the development of MDs, the EU regulations' changes present many challenges to the MD industry. The new stricter (heavy) regulations aim to improve patient safety through more rigorous quality assurance measures, but there is concern that this may adversely affect SMEs.

The Porter hypothesis implies that heavy regulation induces innovation while, on the other hand, there is a contrary view that heavy regulation is a barrier to innovation. Within the context of the new MD development regulations discussed in this paper, it would appear that, with respect to SMEs, heavy regulation may be a barrier to the innovation of new MDs. In contrast, with regard to larger organizations, such regulation may prove to stimulate innovation. However, while some SMEs may move away from MD development to non-MD products, they may transfer their innovative skills to these products to the medical profession's detriment.

Regarding research question Q1, our research shows that the new regulations should ensure improved MD safety. However, the economic load may be excessive both to the producer and healthcare provider. A problem is not only the price of the conformity assessment process, which in the Czech Republic varies with regard to the type and risk class of MD in the range of CZK 150–600 thousand within the assessment according to MDD 93/42/EEC but another difficulty for the manufacturer is the obligation to submit clinical data for conformity assessment. Obtaining clinical data that can be used for MDs' clinical trials and subsequently setting up the PMCF system in clinical trials may also be beyond small companies' power. The evidence also indicates that some SMEs may be forced to diversify to “non-medical” products, with the inevitable loss of some innovative MDs being made available to patients and healthcare providers. Q2 has partly been answered under Q1, but as the number of MD developers reduces so will the number of innovative MDs, thus restricting future MD innovation. Following the introduction of the new regulations, further research should be undertaken to verify our findings and perceived implications for the development of MDs by SMEs. On a more positive note, due to the effect of COVID-19, the implementation of the regulations has been delayed until 2021, giving companies the chance to manage the transition more positively and even seek out benefits through the opportunity of a competitive advantage.

Limitations regarding our study may arise from two perspectives; (i) the paper deals only with the production of MDs subject to MDD 93/42/EC, resp. MDR 2017/745 and does not, therefore, include MDs in class IVD, and (ii) it is based mainly on a single case study – Mediatrade. Due to the specifics of the selected company, namely its unique business model, previously received state subsidies and historical changes in the Czech accounting practices disallow exact replication of this case study. We have addressed this issue by supporting our research with a comprehensive analysis of the whole industry, which confirms the results obtained from Mediatrade. Nevertheless, we believe that the paper provides a valuable contribution to the literature concerning the innovative development of medical devices.

We believe the paper is timely in that it addresses an important current issue regarding the MD industry's future. In further support of the timely relevance of our paper is the concern regarding the current crisis over coronavirus (COVID-19), where innovation in medical devices, especially in the field of personal protective equipment (PPE) and respiratory/intensive care ventilator equipment, should be encouraged and not suppressed through restrictive legislation, although safety must be paramount. This article can also be a basis for further research and addressing issues such as what has happened after that date to confirm the concerns expressed before introducing the new regulations or refute them. How have the SMEs reacted to the new regulations? Has there been a decline in MD innovations? Have some SMEs gone out of business or moved away from MD development? What impact could Covid 19 have on the MD industry?

## Data Availability Statement

The raw data supporting the conclusions of this article will be made available by the authors, without undue reservation.

## Author Contributions

PM, LP, and FL contributed to conception and design of the study. LR performed the statistical analysis and graphical outputs. PM and LH wrote the first draft of the manuscript. PM, FL, LH, LR, and LP wrote sections of the manuscript. All authors contributed to manuscript revision, read, and approved the submitted version.

## Conflict of Interest

The authors declare that the research was conducted in the absence of any commercial or financial relationships that could be construed as a potential conflict of interest.
