# Supplementary material for: Do Regulatory Changes Seriously Affect the Medical Devices Industry? Evidence From the Czech Republic
Source: Front Public Health. 2021 Apr 28;9:666453. doi: 10.3389/fpubh.2021.666453 (PMC8113379; doi:10.3389/fpubh.2021.666453)
Supplement: Supplementary file 2 [file Table_2.DOCX]

**Supplementary File 1**

1. SPSS Output

**Time Series Modeler**

[DataSet3]

| **Model Description** | | | |
| --- | --- | --- | --- |
|  | | | Model Type |
| Model ID | Revenues_Consumption_Material | Model_1 | Brown |

| **Forecast** | | | | |
| --- | --- | --- | --- | --- |
| Model | | 2020 | 2021 | 2022 |
| Revenues_Consumption_Material-Model_1 | Forecast | 114708,64 | 120760,85 | 126813,05 |
|  | UCL | 148118,38 | 160276,00 | 173271,45 |
|  | LCL | 81298,90 | 81245,70 | 80354,65 |

| For each model, forecasts start after the last non-missing in the range of the requested estimation period, and end at the last period for which non-missing values of all the predictors are available or at the end date of the requested forecast period, whichever is earlier. |
| --- |


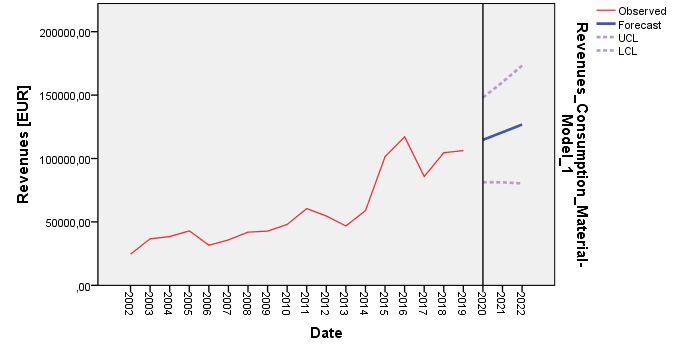


(b) Development of revenues for provided services.

(c) GDP growth and company revenues.
